# Editorial: Management of Patients With Non-dialysis Dependent Chronic Kidney Disease (ND-CKD)

**DOI:** 10.3389/fmed.2021.827245

**Published:** 2022-02-03

**Authors:** Michele Provenzano, Carlo Garofalo, Jose Luis Gorriz, Michele Andreucci

**Affiliations:** ^1^Renal Unit, Department of Health Sciences, Magna Graecia University, Catanzaro, Italy; ^2^Department of Advanced Medical and Surgical Sciences, University of Campania “L.Vanvitelli”, Naples, Italy; ^3^Department of Nephrology, Hospital Clínico Universitario, Health Research Institute of Valencia (INCLIVA), University of Valencia, Valencia, Spain

**Keywords:** chronic renal failure, prognosis, albuminuria, ESKD (end stage kidney disease), cardiovascular risk

Chronic Kidney Disease (CKD) is a clinical condition defined by a reduction in renal function (estimated glomerular filtration rate, eGFR <60 mL/min/1.73 m^2^) and/or the presence of structural abnormalities in the kidney and/or increased (>30 mg/24 h) urine albumin excretion, which persist for at least 3 months (1). Patients with CKD and without appropriate treatment are likely to develop relevant clinical outcomes, such as cardiovascular events (especially heart failure), End-Stage-Kidney-Disease (ESKD), and death (2). The onset of ESKD is often a prelude to the need for renal replacement therapies, such as dialysis or kidney transplant, and, in the patient's perspective, it is certainly an event that marks a substantial change in the quality of life. Owing to this background, great motivation from physicians to improve the diagnosis, treatment, and prognosis of CKD patients is more than expected. With the present Research Topic published in *Frontiers in Medicine* we can definitely confirm our impression. In fact, many authors have examined and provided interesting details around risk factors for the onset of cardiorenal outcomes, pathophysiologic mechanisms active in CKD, and novel therapeutic strategies.

Among the risk factors, the role of intimal and medial vascular calcification (VC) has been revised (Bover et al.). The authors highlighted that prevalence of VC in moderate to severe CKD (G3a-G5 stage) exceeds 50% of patients and that VC is not a unique expression of the mineral bone diseased disorder, but rather a complex clinical entity associated with different endpoints, mainly represented by left ventricular hypertrophy and heart failure for medial VC and endothelial-ischemic damage for intimal VC. Moreover, a combination of calcium and phosphate deposition, in the form of calciprotein particles, and differentiation of vascular smooth muscle cells toward secretory phenotype plays a role in VC development. The implementation of omics techniques in detecting and differentiating VC in CKD patients is a promising and very interesting tool for future clinical application.

Still, in the context of cardiovascular risk in CKD, D'Marco et al. discussed the association between perirenal and epicardial factors with kidney measures (eGFR and urine albumin excretion) and cardiorenal outcomes, such as cardiovascular events, mortality, and eGFR decline. Thus, the authors pointed out the role of these two markers specifically in CKD patients and, interestingly, report the link between adipose tissues, fibroblast growth factor 23 (FGF23), inflammatory cytokines (IL-6), and cardiovascular risk.

Inflammatory cytokines also play a role in the context of diabetic kidney disease (Donate-Correa et al.). Urinary and serum levels of cytokines, like IL-6, IL-8, IL-18, tumor necrosis factor (TNF)-α, and interferon (IFN)-γ which exert pro-inflammatory actions, are increased in these patients and play a significant role in triggering pathophysiological mechanisms, such as inflammation, extracellular matrix accumulation, and fibrosis. More importantly, it has been demonstrated that novel antidiabetic drug agents, namely sodium-glucose cotransporter type 2 inhibitors (SGLT2is) and glucagon-like peptide-1 receptor agonist (GLP-1RA), have a cardioprotective effect, which is partially mediated by anti-inflammatory effect, in addition to other unexpected effects on renal hemodynamics, a decrease of renal and cardiac workload and hypoxia and improving myocardial and renal energetics.

There are few studies demonstrating that treatment with these drugs is followed by a change in inflammatory parameters. However, the development of drugs that specifically target inflammatory pathways and that encourage the use of recent technological innovations, such as antibodies or microRNAs (miRNAs), can really represent a very interesting opportunity for future research. In the context of cardiac markers, in their original article Liu et al. reported that performing coronary angiography within 7 days before cardiac surgery procedures did not modify the risk for the onset of acute kidney injury; that observation should be considered in the preoperative management of high-risk patients.

Obesity and anemia are two important prognostic factors in CKD patients and have been also examined in the Research Topic (García-Carro et al.; Portolès et al.). In both Research Topics, novel interesting future perspectives have been discussed. Regarding obesity, Perticone et al. performed original research by stratifying obese patients into those with Obstructive sleep apnea syndrome (OSAS) and those without OSAS, reporting that the presence of OSAS was significantly associated with a lower eGFR and higher albuminuria levels. Furthermore, a holistic approach has been proposed to manage CKD patients and the results of novel randomized studies testing the efficacy of SGLT2is, GLP-1RA, mineralocorticoids receptor agonists, alone or in combination with classical treatments for obesity are eagerly expected to be obtained. With respect to anemia, a great milestone is represented by the novel Hypoxia-inducible factor prolyl hydroxylase (HIF) inhibitors, which are valid alternatives to the traditional Erythropoiesis-stimulating agents (ESAs) (3). However, Portolés et al. in their article also mentioned on the basis of previous intervention studies that hemoglobin targets in CKD patients should be personalized, this being a valid example of personalized medicine.

Rare diseases are recognized as an important cause of kidney disease and need to be detected as soon as possible in order to plan a specific treatment for each patient. Especially after the demonstration that the cause of CKD (and not only the presence/absence of CKD) *per se* influences prognosis (4). Zhang et al. presented a case series including 19 patients with fibronectin glomerulopathy, an inherited autosomal dominant disease characterized by the presence of proteinuria, microscopic hematuria, and arterial hypertension. All patients enrolled in this study underwent renal biopsy. Interestingly, albeit the sample size was very low, it was still possible to detect subgroups of patients (nephrotic range proteinuria and focal glomerular sclerosis on histology) with a faster renal function decline.

Another inherited disease, the Fabry Disease, has been associated with a very poor prognosis since it causes fast CKD progression, cerebrovascular, and cardiac events. In this volume, Battaglia et al. highlighted the importance of early detection of the disease, which is often misdiagnosed, especially in the pre-dialysis stage of CKD.

Siligato et al. revised the pathophysiology, outcomes, and treatment of nephrotic syndrome due to primary glomerulonephritis (GN) during pregnancy, stratified by type of GN. Based on the accurate literature review summarizing previous studies, those authors found that membranoproliferative glomerulonephritis and focal segmental glomerulosclerosis are associated with a worse prognosis as compared to minimal change disease and membranous nephropathy.

However, this Research Topic seems of great interest with respect to the attitude of nephrologists toward young female patients. Among the category of tubule-interstitial diseases, kidney stones reached a non-negligible prevalence, especially in developed countries. In this Research Topic, Xun et al. developed a predictive model, based on radiomic features and clinical features, for stone-free rate in patients undergoing flexible ureteroscopy. Radiomic refers to computer-assisted techniques of image extraction that optimize the selection of useful features from digital medical images. Other characteristics, which predicted outcome, were stone volume, hydronephrosis level, and operator experience. It is important to note that the authors used an excellent methodology, including least absolute shrinkage and selection operator (LASSO) regression for selecting radiomic features, but also model calibration, model validation, and built a nomogram. These tools are important to apply the clinical finding at the individual level and have been largely evoked for CKD patients (5, 6).

The management of Kidney Transplant recipients (KTR) has made important progress in the past decades, in particular for the knowledge and control of acute rejection (7). Nevertheless, KTRs are at increased risk for long-term graft failure and further effort should be directed toward the comprehension of the underlying mechanisms. Codina et al. have described in detail the causes of damage in KTR focusing on the mechanisms of intrinsic kidney repair that should be improved to reduce the burden of chronic graft failure. Moreover, the role of traditional risk factors of CKD progression, which have been widely investigated in pre-dialysis CKD patients, is less investigated in KTR. The authors have analyzed the effect of the recurrence of glomerulonephritis in kidney transplantation, as well as other factors that may influence the progression of CKD in kidney transplantation, such as drug nephrotoxicity, the post-transplant diabetic nephropathy, and the role of infection and cold ischemia time and ischemia-reperfusion injury. The authors also describe reparation mechanisms of renal lesions in those patients. In a cohort study of 286 KTR, Bielopolski et al. found that microalbuminuria, measured after kidney transplant, was associated with an increased risk for cardiovascular events and death over time, with the association becoming significant after 2 years from albuminuria measurement. This is an important confirmation of what has already been found in CKD patients, where proteinuria is recognized as an independent and strong risk factor for cardiovascular outcomes (8). Thrombotic microangiopathy has been also found as one of the possible complications in KTR (Ávila et al.).

Other than detecting CKD patients at increased cardiorenal risk, there is presently a growing interest in improving treatment and reducing the risk for future events in these patients (9). Novel drugs have been introduced with the aim of reducing the residual risk in CKD patients, on top of the standard-of-care which consists, following the current guidelines, of drugs intervening in the Renin-Angiotensin-Aldosterone system (RAAS) (Provenzano et al.; Shabaka et al.). For instance, SGLT2is have been shown to reduce cardiovascular risk and risk for CKD progression in several recent studies, enrolling both diabetic and non-diabetic CKD patients. Moreover, these drugs share mechanisms of action with a low-protein diet, namely the reduction in hyperfiltration and a correct diet may compensate for the energy-wasting determined by SGL2is, thus improving nephroprotection (Cupisti et al.).

In general, diet has always aroused interest in the nephrology community with the aim of delaying CKD progression in renal patients. Correct incomes in protein, sodium, potassium, calcium, phosphorus, and vitamins should be carefully evaluated both in patients with pre-dialysis CKD and in KTR (Molina et al.). With respect to potassium, a novel interesting therapeutic approach derives from the novel agents reducing serum potassium (sK) levels, namely Patiromer Sorbitex Calcium, and Sodium Zirconium Cyclosilicate, which are able to maintain optimal sK levels without determining hypokalemia and adverse events that were more frequent with old treatments (Morales et al.). The importance of such a finding is not only related to the risk of cardiovascular death due to hyperkalemia but also depends on the evidence that those agents allow to maintain and optimize treatment with RAAS inhibitors and thus can lead to a better prognosis in CKD patients.

Steps forwards have also been made for optimizing the treatment of CKD patients with a concomitant cardiovascular disease. In patients with CKD and concomitant atrial fibrillation, which is a common arrhythmia in CKD, treatment with vitamin K antagonists provided a clinical benefit. However, these drugs have been associated with an increased risk for renal function deterioration as compared with direct oral anticoagulants, which should be accurately tested in patients with advanced CKD (stage 4 and 5) (Cases et al.). The advent of angiotensin receptor–neprilysin inhibitor for the treatment of Heart Failure (HF) has prompted its use in CKD patients given the efficacy in reducing parameters, such as N-terminal pro–B-type natriuretic peptide (NT-proBNP), left atrial size, and the overall risk of death. Fu et al. have shown that sacubitril/valsartan may be effective in patients in CKD stage 5 and HF with a preserved ejection fraction where it reduced NT-proBNP and heart rate while alleviating symptoms of HF, even if further and larger studies should confirm this hypothesis. This Research Topic presents many interesting and stimulating concepts and sets the stage for the future work that is still needed to optimize the management of this complex, yet very interesting disease, CKD.

## Author Contributions

MP drafted the initial manuscript. All authors revised, approved the final version, and agree to be accountable for the content of the work.

## Conflict of Interest

The authors declare that the research was conducted in the absence of any commercial or financial relationships that could be construed as a potential conflict of interest.

## Publisher's Note

All claims expressed in this article are solely those of the authors and do not necessarily represent those of their affiliated organizations, or those of the publisher, the editors and the reviewers. Any product that may be evaluated in this article, or claim that may be made by its manufacturer, is not guaranteed or endorsed by the publisher.
